# Thermal imaging applications in neonatal care: a scoping review

**DOI:** 10.1186/s12884-019-2533-y

**Published:** 2019-10-24

**Authors:** Anastasia Topalidou, Nazmin Ali, Slobodan Sekulic, Soo Downe

**Affiliations:** 1 Research in Childbirth and Health Unit, School of Community Health and Midwifery, Faculty of Health and Wellbeing, University of Central Lancashire, Preston, UK; 2 Department of Neurology, Faculty of Medicine Novi Sad, University of Novi Sad, Novi Sad, Serbia

**Keywords:** Thermal imaging, Infrared thermography, Neonatology, Newborn, Monitoring, Temperature

## Abstract

**Background:**

In neonatal care, assessment of the temperature of the neonate is essential to confirm on-going health, and as an early signal of potential pathology. However, some methods of temperature assessment involve disturbing the baby, disrupting essential sleep patterns, and interrupting maternal/infant interaction. Thermal imaging is a completely non-invasive and non-contact method of assessing emitted temperature, but it is not a standard method for neonatal thermal monitoring. To examine the potential utility of using thermal imaging in neonatal care, we conducted a comprehensive systematic scoping review of thermal imaging applications in this context.

**Methods:**

We searched EMBASE, MEDLINE and MIDIRS.

**Results:**

From 442 hits, 21 met the inclusion criteria and were included in the review. A significant number (*n* = 9) were published in the last 8 years. All the studies were observational studies, with 20 out of 21 undertaken in North America or Europe. Most of them had small cohorts (range 4–29 participants). The findings were analysed narratively, to establish the issues identified in the included studies. Five broad themes emerged for future examination. These were: general thermal physiology; heat loss and respiratory monitoring; identification of internal pathologies, including necrotising enterocolitis; other uses of thermal imaging; and technical concerns. The findings suggest that thermal imaging is a reliable and non-invasive method for continuous monitoring of the emitted temperature of the neonates, with potential for contributing to the assurance of wellbeing, and to the diagnosis of pathologies, including internal abnormalities. However, the introduction of thermal imaging into everyday neonatology practice has several methodological challenges, including environmental parameters, especially when infants are placed in incubators or open radiant warmers.

**Conclusion:**

In conclusion, although the first attempt at using thermal imaging in neonatal care started in the early-1970s, with promising results, and subsequent small cohort studies have recently reinforced this potential, there have not been any large prospective studies in this area that examine both the benefits and the barriers to its use in practice.

## Background

Assessment of the neonatal temperature is important, because normothermia offers reassurance of continued wellbeing, and because both hyperthermia and hyperthermia are associated with underlying conditions that can result in long term irreversible damage, and even death. The issue of neonatal temperature assessment has increased in prominence recently, as concern about sepsis in neonates (both term and preterm) has highlighted the lack of knowledge around hypothermia as a marker of advanced infection [1–6]. The optimum means of ensuring a stable neonatal temperature for a healthy neonate in the first hours of life is to ensure the newborn stays in skin-to-skin contact with the mother, as the physiological maternal response is to thermoregulate with the baby [1–3]. However, for sick term neonates, or for those born preterm, where staying with the mother is not an option, regular assessment of temperature is an essential part of good quality care. Some of the current means of assessing neonatal temperature can disturb the usual sleep/wake patterns of sick neonates, and can even be invasive and distressing [7]. This paper examines current evidence relating to the potential for thermal imaging as an accurate and non-invasive method of continuously assessing neonatal temperature under these circumstances.

### Physiology of fetal and neonatal thermoregulation

The fetal temperature is linked to maternal temperature and to the maternal-fetal thermal gradient. Within the utero-environment, fetal thermoregulation is dependent on the mother, as heat is transferred to the fetus via the placenta and the uterus. In addition, the heat that is generated by the fetus, as a by-product of its metabolism, is eliminated through the mother. The fetal heat production and loss to its maternal surroundings results in thermal stability, with the fetus maintaining a temperature that is 0.3 °C to 0.5 °C higher than that of the mother [1–4].

However, directly after birth, there is a decline in temperature. This is mostly due to the fact that the newborn is exposed to a new environment and has a lack of insulation [2, 8]. Thus, to increase body temperature and prevent heat loss, non-shivering thermogenesis (NST) must occur for the newborn to survive [4, 8]. If NST is insufficient, this poses a risk of the infant developing hypothermia (body temperature below 36.5 °C). This can result in physiological changes occurring within the infant, such as vasoconstriction. Premature newborns are at a greater risk of developing hypothermia, because they have a lower percentage of brown fat in comparison to full-term newborns, since brown fat is acquired in later weeks of gestation [3, 9].

### The case for temperature measurement with thermal imaging

Several new measuring techniques to assess temperature over an extended period have been introduced in the last decade. Some of them involve the use of probes and sensors that require contact which may cause discomfort (especially for the sensitive skin of preterm babies) and mechanical stress, as well as posing possible hygiene risks [7, 10]. Therefore, contact-free and non-invasive techniques are more desirable. One such a technique is thermal imaging (TI) (infrared thermography). TI relies on the infrared radiation that is emitted from the human body. The amount of the radiation that is emitted by any object depends on the temperature of the object and its emissivity [11]. The human body has an emissivity of 0.98 making it almost a perfect emitter [12]. Additionally, the higher the temperature, the more radiation is emitted [11]. The actual exitant radiation from an object or body is described by the emitted, the radiated and the transmitted radiation, the sum of which is always one. Knowing that the human skin is not transmissive but opaque the exitant radiation consists of emitted and reflected radiation [13]. A TI device (camera) can capture images (thermograms) that show thermal distributions of the epidermis in real-time [8, 14]. A thermal image of a human body is a visual representation of the surface skin temperature. However, human skin is a challenging “material”, as the emitted radiation may additionally represent heat transferred from within the body-core structures, as veins, organs, or lumps and inflammations [10, 12, 15, 16]. In newborns, this can be an advantage, however, as TI has the additional ability to efficiently record the thermal energy (heat in transfer) from highly vascular systems, such as heart and liver, by representing warmer areas on the surface, due to the thinner skin and poor insulation [15]. Several researchers have used TI to investigate the body surface temperature, the energy loss and other factors related to neonatal thermoregulation [15, 17–19].

The aim of the current scoping review was to provide a comprehensive overview of the literature regarding the application of TI in neonatal care and outcomes associated with the use of this method, along with an identification of the remaining knowledge gaps.

## Methods

Scoping reviews have been increasingly prevalent in the literature over the last decade or so. Mayes et al. (2001) [20] state that such a review usually: “*aims to map rapidly the key concepts underpinning a research area and the main sources and types of evidence available, and can be undertaken as stand-alone project in their own right, especially where an area is complex or has not been reviewed comprehensively before*”. The methodology can vary, including both qualitative and quantitative research, and it is more iterative and flexible than that standard meta-analytic systematic review approaches. It is usually used either to identify research gaps, or to provide a narrative summary of the field under scrutiny [21, 22]. The protocol for the current review was structured using the scoping review methodological framework described by Arksey and O’Malley (2005) [22].

### Search strategy

An extensive literature search to identify studies published from the earliest date in each database until August 2017 was conducted. Two authors performed a search of the following international databases: EMBASE (1974 to 1st August 2017), MEDLINE (1946 to August 2017 week 1) and Maternity & Infant Care Database – MIDIRS (1971 to July 2017). In order to find all the related articles, we used sensitive search strategies to reduce the risk of losing any articles. The search was designed to retrieve all articles combining the concepts of “thermography”, or “thermographic”, or “thermal imaging”, or “long-infrared” and “infant”, or “newborn”, or “neonatal”, or “neonate”. The search strategy was limited to the English language only. The above sources and databases were considered by the researchers as the most suitable for the objective of the study, as we thought they would include the majority of published papers in the general field of interest.

### Inclusion/exclusion criteria

We did not assess the methodological quality of the included studies in this scoping review, as the purpose was to scan the literature and to determine what has been reported and what needs further investigation. All research studies reporting the use of TI in newborns were included. We used the World Health Organisation (WHO) definition of ‘newborn’ [23]: “*A newborn infant, or neonate is a child under 28 days of age*”.

Publications presenting studies of newborns older than 28 days, or where the age was not specified, were excluded. Papers that were not available in full text, that were not focused on healthcare, and that did not report a research or case study with clearly defined methods and results, were also excluded.

### Screening and charting

Duplicate articles were automatically identified by the search tool and removed from the database prior to screening. A two-step process with two independent reviewers for each step were performed. At the first stage, the titles and abstracts of articles identified by the search strategy were screened according to the inclusion and exclusion criteria described above. Disagreement between reviewers during this screen resulted in the article’s inclusion for full-text review.

Studies meeting the criteria outlined were charted using Microsoft Excel and screened further as full manuscript. The full texts were assessed, at this stage, for final inclusion in our study. When the final number of the records selected to be included in the current study were identified, an additional check was performed on the bibliographic reference lists of these articles, to identify any additional eligible publication.

### Analysis

A narrative analytic approach was taken, based on key principles from meta-synthesis theory [24]. First, two authors independently read four of the included papers, and charted the themes they observed. These were then agreed by consensus. The rest of the data set were then read by the same two authors independently and the data were mapped to the initial themes. Any data that could not be mapped to these themes were coded into new themes, and the process continued iteratively. The final thematic structure was agreed between all the authors.

## Results

**Fig. 1:**
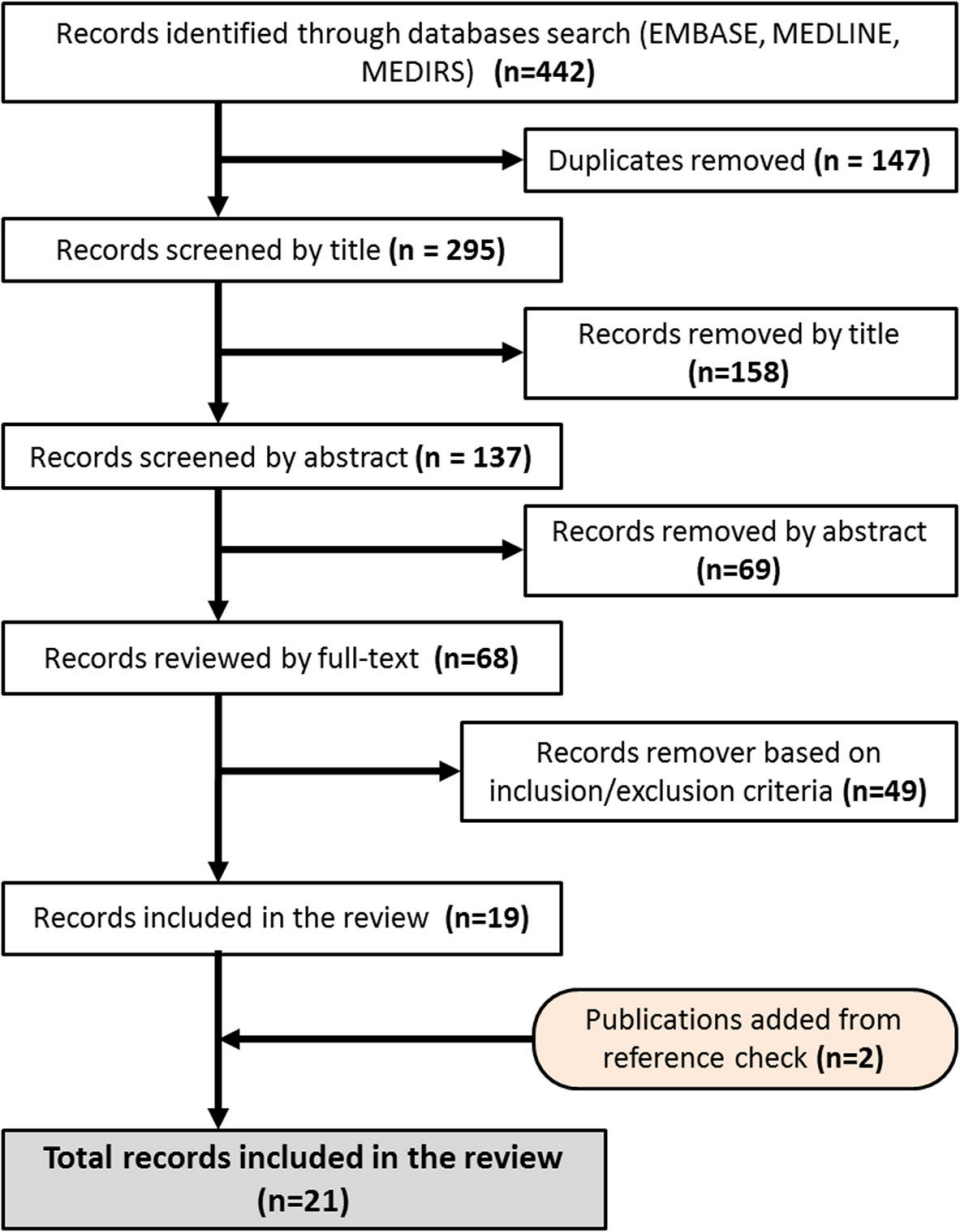
Flow chart of literature review process

A total of 442 records were identified by the search strategy of the databases (EMBASE, MEDLINE and MIDIRS). After de-duplication 295 records were screened by title in order to make the procedure more manageable. Overall, 137 records were screened by abstract, and 68 were taken forward for full text review. After the final full-text selection, 19 records remained and were included in this review. In two of these [25, 26] the age of the neonates was not explicitly defined as a number, but the text indicated that they were eligible, and so they were included. Checking the references of included papers resulted in two additional included studies. The screening process and final included studies are documented in the flow diagram (Fig. 1).

**Fig. 2:**
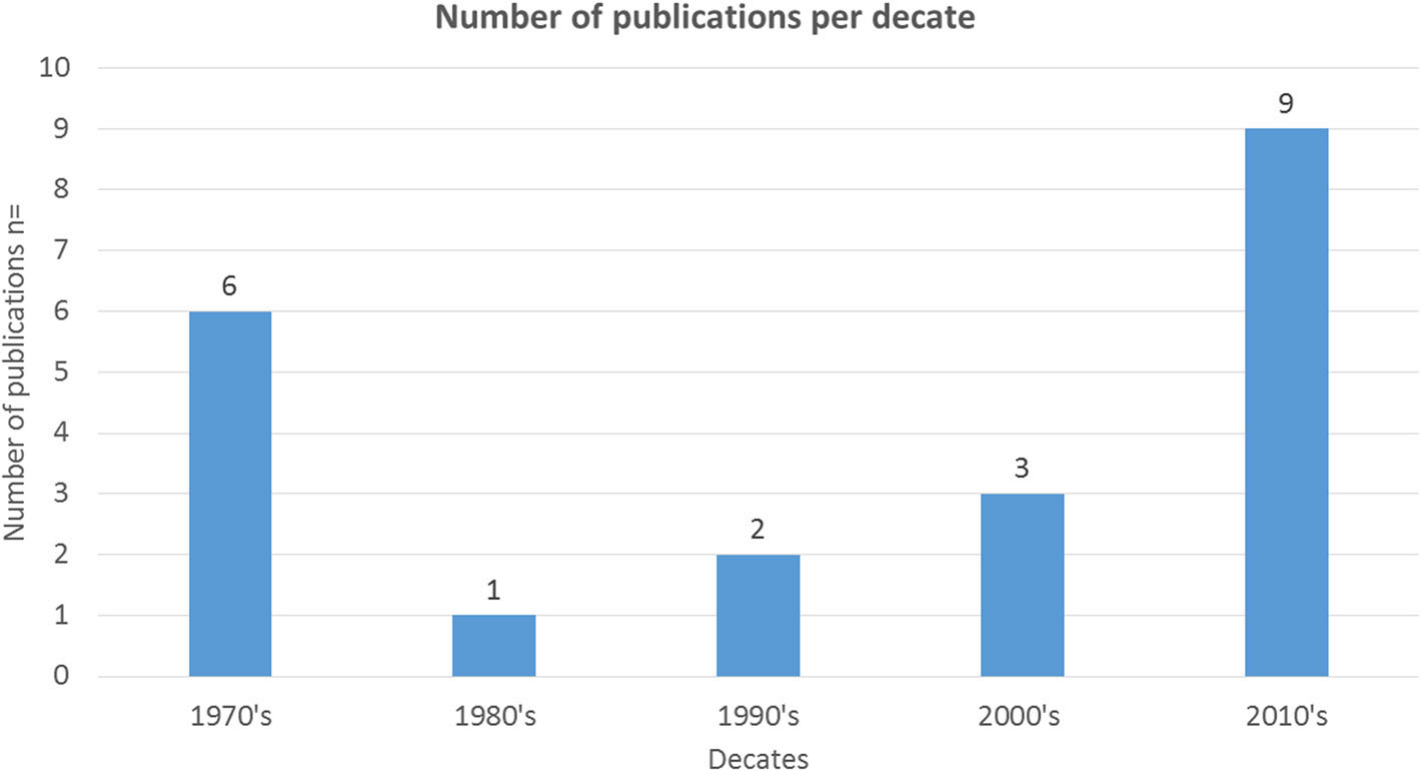
The number of publications per decade

The characteristics of the 21 included studies are presented in. All were observational studies. The twenty one of them were case series and one was a comparative cohort study. Although the first attempts to use TI in neonatology started in the early-1970s, two chronological gaps, with no papers published, were observed. The first gap was between 1980 and 1997 and the second one between 2003 and 2008, after which interest seems to be rapidly rising. The number of publications per decade are presented in Fig. 2.

The included studies were undertaken in the following countries: USA (*n* = 9 studies), Austria (*n* = 3), Germany (*n* = 2), France (*n* = 1), Sweden (n = 1), Finland (n = 1), Sweden and Finland (*n* = 1), Japan (n = 1) England (n = 1), and Canada (n = 1). They generally included small samples, ranging from *n* = 4 infants [35], to *n* = 29 preterm neonates assessed on night 9 of life [39]. Although, there were two studies with larger cohorts of 41 [17] and 43 newborns [29], both of them had their cohorts divided into 4 smaller sub-groups with different characteristics. Some studies included larger cohorts [15, 25], but it was not clear how many of the assessed newborns were < 28 days of age (Table 1).

**Table 1:** Final records included in the scoping review based on the inclusion and exclusion criteria (in chronological order)

Author(s)	Year	Type of Study	Country	Purpose of the Study	Population	TI Assessment Tool
Viitanen & Kivikoski [27]	1971	Case series	Finland	To assess the thermographical changes in the temperature of the newborns and the comparison with local skin temperature measurements.	18 newborns (immediately after delivery)	AGA Thermovision system Model 652
Tahti et al. [28]	1972	Case series	Finland & Sweden	To record the emitted heat of wide areas of infant’s body and to study the infant’s first reaction to a cold environment.	16 infants directly after delivery (12 healthy full-term infants of normal weight; 2 healthy premature infants with 1800 g and 2200 g body weight; and 2 asphyxiated babies with 1800 g and 2600 g birth weight)	AGA Thermovision system Model 661
Rylander et al. [29]	1972	Case series (with 4 groups)	Sweden	To demonstrate if a cold-induced increase in heat radiation appeared over areas where brown fat should be subcutaneously situated.	43 healthy infants (gestational age 38–44 weeks) checked before the tenth day of life Group 1, *n* = 19: infants with a birth weight of 2750 g to 3820 g (in environmental temperature of 24 °C) Group 2, *n* = 10: infants with a birth weight of 3240 g to 4020 g (placed in an incubator) Group 3, *n* = 7: infants with a birth weight of 1860 g to 2680 g (treated in the same way as Group 2) Group 4, n = 7: infants with a birth weight of 2980 g to 4370 g (in a water bath of 38 °C)	AGA Thermovision system Model 660
Perlstein et al. [30]	1972	Case series	USA	To investigate if infant age is an important variable to consider in evaluating interscapular skin temperatures in cold-stressed babies.	14 full-term and premature infants (7 of the infants were less than 24 h and 7 more than 5 days old at the time of examination)	No brand name (thermography system Barnes Engineering Co, with an Indium Antimonite sensor 2–4.4 μm. 4 thermograms per second)
Bhatia et al. [31]	1976	Comparative cohort study (follow up of patients’ group sub-sample)	USA	To investigate if TI can be informative in acute and chronic liver disease, particularly on follow-up basis.	Patients group: 62 infants and children from 3 weeks to 17 years of age Control Group: 32 healthy children from 2 days to 8 years of age (no specification on how many of them were newborns < 28 days of age) Follow-up was performed in 28 of the 62 participants (patients group)	AGA Thermovision system (no model description)
Pomerance et al. [15]	1977	Case series	USA	To determine normal anterior and posterior thermograms of the trunk of a newborn; and to investigate whether deep-lying organs can be detected at the surface.	37 newborns (age range not specified for whole group). There was one 2-day-old term, one 3-days old term, one 18 days old pre-term, one 19 days old pre-term etc.	Spectrotherm 2000 Thermographic System
Clark & Stothers [14]	1980	Case series	England	To visualise skin temperature distributions in newborns; and to compare temperatures obtained from thermograms (thermal camera) to skin temperatures measured with a thermometer.	17 newborns 4–13 days old(15 were full-term and 2 were preterm).	AGA Thermovision system Model 680
Oya et al. [32]	1997	Case series	Japan	To measure the extent of non-shivering thermogenesis (NST) in brown adipose tissue of human newborns receiving routine thermal care and to examine the influence of oxygen levels at birth on the initiation of NST.	15 healthy full-term newborns (five minutes after birth)	Thermal Video System 3000 ME, Japan Avionics Co.
Ek et al. [18]	1999	Case series	USA	To investigate the changes in heat loss when radiant warmers were removed and returned to premature infants.	10 premature infants (gestational age 31.4 ± 5.5 weeks), age 15 ± 11.7 days	600 L infrared imaging radiometer (Inframetrics)
Adams et al. [19]	2000	Case series	USA	To test a new method – infrared thermographic calorimetry – against respiratory indirect calorimetry to measure mean body temperature and calculate heat loss.	10 preterm infants (34 ± 23 days)	Inframetrics model 525 infrared camera
Christidis I et al. [17]	2003	Case series (with 4 groups)	Austria	To investigate a surface temperature profile in newborns within the first hour after delivery.	41 newborns (within the first hour after birth) Group 1, *n* = 19: infants after normal pregnancy, wrapped into cotton immediately after delivery Group 2, *n* = 15: infants examined by paediatrician under a radiant heater Group 3, *n* = 4: infants after normal pregnancy who had skin-to-skin contact. Group 4, *n* = 3: infants after normal pregnancy, recorded before any intervention.	Thermotracer TH 3100 (NEC San-ei Instruments, Japan)
Saxena & Willital [26]	2008	Case series	Austria	To assess the application of TI to identify pathologies in 1 week to 16 year old children. (In newborns with abdominal wall defect)	285 patients; 18 newborns (> 1 week old)	Talytherm thermal imager (Rank Taylor Hobson Ltd)
Rice et al. [33]	2010	Case series	USA	To measure the abdominal surface temperature in low birth weight newborns, using thermography, and drawing comparisons between abdominal and thoracic surface temperatures in newborns with and without necrotising enterocolitis (NEC).	13 newborns; 10 newborns had radiographs and were used for comparison. (23–29 gestational weeks; examined during the first month of life)	FLIR SC640 camera
Herry et al. [25]	2011	Case series	Canada	To compare thermograms of the abdomen of healthy newborns and newborns with NEC, to distinguish differences and to investigate if TI is suitable for diagnosing NEC in infants.	59 newborns (48 were had a gestational age of 28.3 ± 2.4 weeks; 11 were of 26.7 ± 1.8 weeks)	No brand name (Infrared camera, uncooled microbolometer focal plane array, 320 × 420 pixels, thermal and spatial sensitivity of 0.05° at 30 °C and 1.3 mrad)
Abbas et al. [34]	2011	Case series	Germany	To use TI to monitor thermal distributions of neonates within the neonatal intensive care unit.	7 preterm newborns (gestational age was a mean of 29 weeks, included in the study directly after birth)	VarioCAM hr. head camera (InfraTech GmbH)
Knobel et al. [8]	2011	Case series	USA	To measure body temperature in infants and examine the relationship between body temperature and symptoms of NEC in infants with low birth weights.	10 low birth newborns (gestational age less than 29 weeks, examined during the first 5 days of life)	FLIR SC640 uncooled infrared camera
Knobel et al. [35]	2013	Case series	USA	To test instrumentation and develop analytic models to use in a larger study to examine developmental trajectories of body temperature and peripheral perfusion from birth in extremely low birth weight (EBLW) infants.	4 newborns, 4 h of birth (< 29 weeks gestational age)	Not mentioned
Heimann et al. [36]	2013	Case series	Germany	To evaluate skin temperature by using different positions with TI in multiple body areas of preterm infants for detailed information about temperature regulation and distribution.	10 preterm infants (12–62 days old)	VarioCam hr-Head (InfraTec GmbH, Germany)
Kurath-Koller et al. [37]	2015	Case series	Austria	To evaluate the safety of laser acupuncture in newborn infants by using a thermal camera to analyse changes in thermal distributions.	20 newborns (23 days old)	FLIR i5 camera
Knobel-Dail et al. [38]	2017	Case series	USA	To explore the utility of TI as a non-invasive method for measuring body temperature in premature infants in an attempt to regionally examine differential temperatures.	Data was collected from 31 infants originally; only 22 had valid thermograms and the first two were used for training (23 to 28 gestational weeks; first 5 days of life)	FLIR SC640 uncooled microbolometer
Barcat et al. [39]	2017	Case series	France	To investigate whether or not skin temperature and vasodilation of the skin affect sleep propensity in neonates.	29 preterm newborns (9 days old)	B400 FLIR Systems infrared camera

The techniques and cameras used progressed rapidly through the years. The early studies [27–29, 31] used the very first commercial infrared system named AGA Thermovision (by AGA, Lidingo, Sweden), which was available on the market before the mid-1960’s. Models such as the 660 and 661 had a single element liquid nitrogen-cooled indium antimonide (InSb LN2) detector, and field of view (FOV) 5^o^x5^o^, with the camera alone weighing 25 kg. The total weight of the whole thermographic system was about 75 kg [40]. In 1980, Clark and Stothers [14] used the AGA Thermovision 680 that was utilised for medical applications. Although it was lighter than the earlier models (15 kg), due to its size and weight it was usually operated on a tripod. Other early studies (1977) used the Spectrotherm 2000 by General Electric, which was equipped with a single element liquid nitrogen cooled Mercury Cadmium Telluride (HgCdTe) detector, with a resolution of 256 × 256 points, and the need for conversion and use of a Polaroid film [15]. The latest studies used the much more sophisticated high resolution radiometric thermal imagers, such as the FLIR SC640 uncooled camera (1.8 kg) with thermal sensitivity of <30mK, resolution of 640X480, FOV ranging from 12°× 9° to 45°× 34° and radiometric video recording with real-time analysis (2017) [38].

The areas of interest that have been investigated to date with these technologies are presented in Table 2.

**Table 2:** Summary of the regions of interest that were assessed up to date

Regions of interest/investigation	Authors - Studies
Body surface (skin) temperature, and temperature distributions (patterns): Forehead, nose, cheeks, chin, earlobe, nape, interscapular area, hand, foot, upper trunk, buttock, thigh, calf, arm, abdomen, back.	Viitanen&Kivikoski (1971); Tahti et al. (1972); Rylander et al. (1972); Perlstein et al. (1972); Bhatia et al. (1976); Pomerance et al. (1977); Clark &Stothers (1980); Ek et al. (1999); Oya et al. (1997); Adams et al. (2000); Christidis et al. (2002); Saxena&Willital (2008); Rice et al. (2010); Herry et al. (2011); Knobel et al. (2011); Abbas et al. (2011); Knobel et al. (2013); Kurath-Koller et al. (2015); Knobel-Dail et al. (2017); Barcat et al. (2017)
Deep structures/organs: heart, liver and kidneys.	Bhatia et al. (1976); Pomerance et al. (1977)
Clinical states: Pathologies, abdominal wall defects, NEC, heart failure, liver diseases, kidney dysfunction.	Bhatia et al. (1976; Pomerance et al. (1977); Saxena&Willital (2008); Rice et al. (2010); Herry et al. (2011); Knobel et al. (2011)
Heat loss	Tahti et al. (1972); Ek et al. (1999); Adams et al. (2000)
Respiratory monitoring	Adams et al. (2000); Abbas et al. (2011)
Safety of laser acupuncture	Kurath-Koller et al. (2015)
Sleep propensity	Barcat L et al. (2017)

The narrative analysis generated five broad themes: general thermal physiology; heat loss and respiratory monitoring; identification of internal pathologies; other uses of TI; and technical concerns.

### General thermal physiology

Since the beginning of 1970’s researchers used thermography as a method to map out the heat distribution of the skin. In 1971, a study assessing the skin circulation changes in newborns, showed differences in the thermal patterns and the level of temperature rise between the trunk and the colder limbs. These patterns were not constant in infants whose mother had pre-eclampsia in comparison with those who did not even though all the births were vaginal in this cohort. In addition, this study showed that thermograms and skin temperature curves both recorded similar data for changes in postnatal temperature [27].

Continuous recording of heat emission of wide areas of the newborn, was reported by the Tahti et al. [28]. In the same study, the temperature change of the umbilical cord was recorded, showing that the temperature of the umbilical cord dropped instantly in infants born with nuchal cord but took 30–60 s for infants born without nuchal cord. Rylander et al. [29] recorded the dorsal surface of infants during 30 min of exposure to an ambient temperature (21-23 °C), reporting no relation to the condition/treatment before exposure and no significant differences between the four observational groups (Table 1). In another study, the age-related thermal adaptive phenomenon in infants was described, following the recording of the interscapular area, which showed that temperature was maintained even under cold-stress conditions [30].

In 1977 Pomerance et al. [15] conducted a study using TI to determine the typical anterior and posterior views of the trunk of a newborn. Over three months, anterior and posterior thermograms of 37 newborns were recorded. Results demonstrated that the heart, liver and kidneys were warm areas due to being highly vascular organs. Warm areas were also observed in the neck, near the umbilical and areas that have high percentages of brown fat. An infant who developed congestive heart failure demonstrated a cooler chest area. A thermogram of another infant revealed a warm area over the left kidney – but not over the right, suggesting limited blood supply to the right kidney. This was later confirmed through autopsy. This study proved that TI has many potential uses within neonatal care and can be used to screen newborns for internal abnormalities, as deep-lying structures in newborns have less insulation.

Three years later, in 1980, Clark and Stothers [14] analysed temperature distributions of infants in different environmental conditions ranging from 28.5 °C to 32 °C. Infrared colour thermography was used to determine the skin temperature of seven infants. In addition, multiple recordings of skin temperature, using an Ellab Universal Thermometer with a probe, were then taken from twelve infants. They observed that, within a warmer environment, the upper anterior region of the neonatal trunk was at a higher temperature in comparison to the posterior region. It was also observed that there was a decrease in temperature in the extremities. In cooler conditions, hot spots were identified over the jugular vein and regions of the carotid artery.

Almost 20 years later, Oya et al. [32], used TI to assess the extent of NST in brown adipose tissue of infants receiving routine care. The findings of this study came in line with earlier findings [29, 30] showing that the interscapular area was warmer than other parts of the body and that NST is activated soon after birth, proving that TI could be useful for the detection of NST in subcutaneous brown adipose tissue.

Another area of research has been skin-to-skin care (SSC) in premature newborns. Knowing the various positive effects of SSC, in 2012 TI was used to assess the skin temperature, distribution and kinetics, to clarify temperature regulation longitudinally. Results showed that during SSC premature newborn’s average skin temperature increased and then dropped down after placing back into the incubator, to a level that was significantly lower, compared to the initial recording. Premature infants needed more that 10 min to regulate their temperature back to the original level. As daily routine care is performed directly after the infants’ placement into the incubator, the above finding is of clinical significance [36].

### Heat loss and respiratory monitoring

The first attempt to record heat loss and infant’s reaction to a cold environment was made by Tahti et al., in 1972 [28]. By using an early model of an infrared camera in combination with an electrical thermometer, the authors found that premature infants showed fairly uniform and similar patterns of change in heat emission as full-term infants. A quick drop of temperature starting from the periphery and rapidly advancing in centripetal direction was observed. During delivery, once the fetal face appeared, its temperature decreased by 2 °C within 10 s. Also, the skin temperature of the anterior thoracic area dropped instantaneously with the first deep breath. On the other hand, asphyxiated babies showed a different pattern, with the temperature of the face, hands and legs dropping, but the temperature of the trunk remaining warm, until the establishment of the respiration. Almost three decades later, in another study, assessing the body surface temperature profiles in four groups of infants within an hour after birth, the authors reported similar patterns of heat change, with the peripheral sites becoming cooler more quickly, in contrast with the trunk. In addition, the author reported that skin-to-skin contact is an effective method to prevent heat loss [17].

Ek et al. [18] studied 10 premature infants to observe what changes would occur when they were removed from radiant warmers. Infrared thermographic calorimetry was used. This method is a combination of a thermal camera and equations that calculate heat expenditure. Using computer software, mean body surface temperatures (MBST) were calculated. They observed that once the radiant warmer was removed, there was a large increase in heat loss and MBST decreased. It was also found that there was a correlation between temperature and age, as the youngest newborns had greater declines in MBST and a larger increase in heat loss. Similarly, a year later, Adams et al. [19] conducted a study using infrared thermographic calorimetry to examine energy loss in 10 preterm infants. Respiratory indirect calorimetry was also used to draw comparisons. Both methods were used to determine the mean of the infants’ body surface temperature. Results showed that there was no significant difference between the mean values obtained from both methods. The authors concluded that the use of TI to calculate energy loss was promising. In another study, real-time TI recording was used to monitor rate of respiration in seven premature neonates (2 in warmer beds and 5 in incubators) and similar results were obtained. Specifically, there were temperature changes within the nasal area (the maximum change being 0.66 °C) indicating that TI could be used in intensive care units to monitor respiration and heat loss of neonates [34].

### Identification of internal pathologies (particularly, necrotising enterocolitis)

TI appears to aid in the diagnosis of pathologies in neonatal internal organs. The first report was published in 1976. In this study, researchers investigated the application of TI in the study of liver diseases. The results showed that specific patterns of heat emission over the liver area were predominant in the participants who had a hepatic disease, such as neonatal hepatitis. Although the findings were not helpful in differential diagnosis between the different liver diseases and conditions, the authors stated that TI could be useful in follow-up investigations, due to its ability to identify changes in thermal patterns [31]. In addition, as presented above, Pomerance et al. [15], demonstrated that pathologies of highly vascular internal organs, as heart and kidney might be detectable by TI.

In 2008, Saxena and Willital [26] conducted a study that evaluated the application of TI to diagnose pathologies in children aged 1 week to 16 years old. Participants included 18 neonates with abdominal wall defects (gastroschisis or omphalocele). Due to the open abdomen solvent-dried dura implants were applied. During the first week the area that was covered with the patch presented lower temperatures in comparison with the skin of the abdominal wall. Four weeks after implantation and until complete implant integration the temperature of the patches increased, appearing higher than the abdomen’s skin, demonstrating the vascular proliferation and the patch integration. The same participants were followed-up a year later showing no significant temperature differences on their abdominal wall.

Herry et al. (2011) [25] studied 48 healthy newborns and 11 newborns diagnosed with necrotising enterocolitis (NEC) to distinguish thermographic differences that would aid in the diagnosis of NEC in future. The surface temperatures of different areas of the abdomen were measured using a thermal camera at a distance of 60 cm. It was found that over a period of time there was a general decrease in temperature in both groups. However, the NEC group had a lower decrease in comparison to the healthy group, possibly due to the increased amount of heat emitted from inflamed bowels. This finding suggests that NEC newborns can be differentiated from healthy newborns, and that TI can aid in the diagnosis of NEC.

This finding is reinforced with a study assessing the use of TI in low birth weight newborns to measure abdominal skin temperature and to assess the relationship between abdominal skin temperature and NEC [33]. In total, 10 infants aged 23 to 29 gestational weeks (of 13 in the cohort) were examined to assess the correlation between temperature and NEC. These infants had both thermograms and radiographs of the abdomen. Temperatures of the abdomen and thorax were measured, and means were calculated. Axillary temperatures were also taken by a probe to measure the accuracy of TI. Those that had been diagnosed with NEC had lower abdominal temperatures in contrast to neonates without NEC [33]. Similar results were also found by Knobel et al. (2011) [8]. They conducted two pilot studies: one studied the feasibility and the methods of using TI to record the body temperatures of neonates in incubators during their first 5 days of life, and the other examined the correlation between body temperatures of 10 extremely low birth weight neonates and the possible development of NEC. Infants were held in the supine position whilst the thermal camera was used. Mean temperatures of the abdomen and thorax were calculated to determine if there was an association with NEC. In the 10 premature neonates, it was observed that those with a diagnosis of NEC (*n* = 10) had lower abdominal temperatures, agreeing with the results from Rice et al. 2010 [33] and Herry et al. 2011 [25]. Furthermore, the pilot studies confirmed that TI is a safe and completely non-invasive method to be used on premature infants with low birth weights, while being able to be used for continuous daily imaging and temperature recording.

Some years later, Knobel-Dail et al. (2017) [38] investigated the accuracy of TI in comparison to using a probe to measure body temperature, and the possibility of using TI to detect early perfusion injury signs, such as NEC in premature neonates. Temperatures of the abdomen and foot were measured using probes, while a thermal camera was used to take images of the neonates within their first five days of life. Thermograms were obtained from 22 infants; the first two infants were used for training research assistants in TI. It was found that mean temperature of the abdomen was 36.44 °C when using a probe, whilst it was recorded to be 36.57 °C using TI showing a good level of agreement. Agreement of the mean temperatures of the foot measured by the two methods was not as good. However, the mean difference was still centred around 0. This demonstrates that TI has potential in aiding in diagnosing and caring for newborns with NEC.

### Other uses of thermal imaging

TI has been used to assess the relationship between skin temperatures, vasodilation and sleep propensity in premature newborns. In a study conducted by Barcat et al. (2017) [39] twenty nine premature newborns (9 days old) were observed. Sleep propensity was defined as occurrences of wakefulness; these were measured alongside thermal recordings of abdominal and distal regions (foot, thigh and hand). The differences between the temperature measurements were defined as peripheral vasodilation. Results showed that shorter durations of sleep propensity correlated with higher temperatures of distal areas and the chest, associated with moderate vasodilation within the areas mentioned. The authors concluded that distal skin temperatures influence sleep onset in premature infants.

Kurath-Koller et al. [37] studied thermal changes that occurred during laser acupuncture to evaluate the safety of laser acupuncture in neonates. Overall, 20 infants, with a mean age of 35 gestational weeks, were studied. Before laser acupuncture was performed, thermograms were taken of both hands. Vital signs were also recorded. An increase in temperature was observed on both hands. The maximum temperature measured was 38.3 °C for the right and 38.7 °C for the left hand. Vital signs showed no significant change. As none of the neonates had significant differences in thermal distributions, it was concluded that acupuncture is safe to be used on neonates. Nonetheless, the authors note that warming of skin during or after the procedure ought to be treated with care.

### Technical concerns

Despite the increasing use of TI applications in neonatal care, thermography has several methodological challenges. In a study conducted in 1997, the infants were placed in an incubator, in order to maintain thermal stability during consecutive recordings. To overcome the barriers created by the materials that could intervene between the camera and the infant, researchers made a 10x15cm window in the canopy. To avoid any heat loss and influences of ambient temperature, the window was sealed with a polyvinyl cloth that enclosed the camera [32]. In 2013, in a study where the maturation of body temperature control in preterm infants was investigated with different temperature instruments, some specific challenges were reported, as they assessed infants housed in plexiglass incubators [35]. The sheet of polyurethane that was used to prevent heat loss resulted in a blurred image, and therefore had to be replaced with a plastic sheet that was secured with tape. In this study, valid and reliable measures were obtained, so the authors concluded that a well-structured methodology with an appropriate camera for this specific environment could provide beneficial research opportunities related to body temperature and peripheral perfusion from birth in extremely low birth weight infants [35]. Other reported difficulties included water droplets on the polyurethane sheet, the type of camera, which was not cordless and had to be connected to a laptop, the limited lens depth and the small port-hole of the incubator [8, 35, 38]. In addition, although an ideal condition is a newborn that is perpendicular to the line-of-sight, achieving this is not always possible. Optimising the viewing angle condition and the selected field of vision, taking account of the wellbeing of the newborn and any clinical imperatives, is an important consideration for correct measurements [41, 42].

## Discussion

This scoping review provides an overview of current knowledge relating to the potential for TI applications in neonatal care. Our search was limited to the English language only, as no funds were available for translation. Excluding languages other than English may introduce a language bias and lead to erroneous conclusions, and such exclusions are not part of our usual practice when undertaking systematic reviews. However, translation of technical data from basic science and technical studies is likely to be more complex than for other types of studies, and there is no a priori reason to assume that technical studies carried out in different languages would generate different results.

All the studies but one that were eligible were case series or observations on a series of infants. In the only study that was described as a comparative cohort study, the age variable of the patients groups was not similar to control group, which means that the participants assigned to the two groups were not representative of the same population. Most of the studies had small cohorts or limitations of case reports’ intrinsic methodological problems, while others presented heterogeneous samples. Despite the generally promising nature of TI results since the early seventies, a large-scale study or a well-designed comparative cohort study appears to be missing from the current literature (Table 1). The range of health care facility conditions in the included studies means that is impossible to assume that the same recording environment was present for all the infants. Also, there were differences between the age of the neonates, their underlying conditions, types of incubators and radiant warmers [25, 34, 38].

An infant exchanges heat with the environment via the respiratory tract and the skin. Five of the included studies demonstrated that TI is a promising method for the continuous monitoring of respiration and the energy/heat loss, especially in intensive care units, opening the road for further studies and adding new knowledge in the field of neonatal monitoring [17–19, 28, 34]. However, as these studies had either a small sample size (max *n* ≤ 16 [28]), or the examination was conducted in sub-groups [17], a further investigation in a bigger cohort, with a standardised protocol and homogeneity in the procedures and participants is needed.

TI showed very good results in the investigation of mother/infant skin-to-skin physiological effects, by providing excellent results while being totally non-contact [17, 36]. As skin-to-skin has been proposed as an optimal approach for keeping premature babies heat stable [36], TI could be a method that will offer an in-depth understanding and facilitate further research in the field.

Additionally, TI showed good results and future potential as a method of continuous surveillance of neonates and their body temperatures for the identification and monitoring of NEC, which is one of the most common neonatal gastrointestinal emergencies, with a mortality rate of 15–30% [8, 25, 33, 38]. The imaging methods currently used for this purpose (abdominal radiography and abdominal ultrasonography) have several limitations and do not always detect early or subtle signs of NEC. The gold-standard abdominal radiography lacks sensitivity and specificity to the changes seen in NEC, especially in early stages [25]. Oh et al. [43] published an overview of several non-invasive technologies that has been used or tested for the detection of NEC (magnetic resonance imaging, gastric tonometry, pulse oximetry, near infra-red spectroscopy etc.), stating that all of them have significant limitations and further adaption is required. Herry et al. (2011) [25] proposed a methodology that revealed very good results, by using abdominal temperature to classify healthy infants and those with NEC, reporting a correct classification rate of > 90%. Other studies suggested that the in-depth understanding of skin temperature and perfusion may provide a better insight into the pathophysiology of NEC [8, 33, 38]. Additionally, the early diagnosis and continuous observation of body temperature differences in neonates could significantly decrease the length of hospitalisation and medical cost [8].

Since the infant’s internal organs are poorly insulated, almost forty years ago, Pomerance et al. [9] and Bhatia et al. [31] reported that TI has the potential to identify pathologies in highly vascular deep-lying organs like the heart and the kidneys [9] and being used for follow-up examinations in liver diseases [31]. Although the findings were promising, and it might be especially relevant for premature newborns, whose body fat is lower, these observations were not pursued again by researchers until there was a sudden surge of interest/publications between 2008 and 2011 [8, 26, 32, 37]. Nevertheless, no similar studies were identified since 1977. One reason for this could be that TI cameras used to be very expensive and the method has not generally been popular in the medical field, as it was mainly used in industrial and military applications [8, 26, 44]. A reduction in the cost of the cameras, and an increase in quality has led to an upsurge of interest in TI in medical applications. From bulky devices and gray scale low resolution images, the technology has moved to affordable and portable high speed and resolution cameras, with thermal sensitivity (NETD-Noise Equivalent Temperature Difference) of <15mK in some cooled cameras. Additionally, radiometric images provide the opportunity for sophisticated analytics. As a result, and knowing that TI is a completely non-invasive method there has been a very recent renaissance of interest for using this method in medical applications [44]. It is worth mentioning that early cameras were not able to record temperature values, and therefore researchers had to use a separate thermometer (usually a skin thermometer) to record temperature values in combination with an infrared camera to make thermal images (recordings of infrared emission variations). In addition, to display the images, researchers had to use a polaroid photographs or similar photographs and usually the analysis was performed with a densitometer using a standard black body scale [27–30]. Recent developments in TI equipment have overcome these problems, meaning that this area could now be very ready for research investment.

Despite the described developments, TI applications in neonatology can be more technically challenging that in other medical fields. The particular barrier is the use of radiant warmer and incubators, as other heat sources or materials with high reflectivity can affect the recordings [14, 18, 45]. However, radiant warmers and incubators are essential, as they secure the maintenance of a thermally stable environment, which is a key task in neonatology [2–4, 8, 9, 18, 38, 41]. To accommodate the environmental confounders, the main methods for the assessment of a neonate’s body temperature when they are in a controlled warmed environment include either cable-bound sensors or sensors that require skin contact. The potential for mechanical stress has led clinicians and researchers to investigate cable-free, non-contact and non-invasive methods [7, 10, 38, 42], and this is the context in which TI has been tested as a potential solution. Two studies describe in detail the methodology behind real-time TI assessment, highlighting the importance of the environment, settings and external sources for accurate recordings [41, 42]. Both studies concluded that a more reliable measurement protocol is needed.

During the last years, infrared windows have been developed allowing the inspection or recording of closed areas. This can be a solution to the described difficulties in comparison with the reported improvised windows [32, 35]. The infrared windows comprise Calcium Fluoride (CaF_2_) lenses (or other materials) that are able to transmit short, mid and longwave IR waves, and can be easy installed on any surface [46, 47]. The infrared windows are the perfect solution for incubators, proving a huge advantage and step change in development in TI applications in neonatology, as they keep the environment enclosed, and allow a TI camera to record continuously.

## Conclusion

A thermally stable environment and continuous body temperature recording are crucial factors in neonatology, for sick or preterm infants. This review demonstrates that TI has potential for further development in the field. Apart from the continuous measurement of the skin temperature and the ability to provide visual thermal maps from major body parts, TI appears to be able to detect pathological conditions that include inflammatory responses. Despite, the almost 50 years of research on the application of TI, and the generally positive results obtained, research studies in big cohorts using established methodological protocols are missing from the literature. The latest rapid development in thermal cameras, image analytics and other components offers great potential for future TI application in neonatology.

## Data Availability

All articles retrieved through standard university searches. All data used are presented and included within the manuscript.

## Abbreviations

FOV: Field of View
MBST: Mean body surface temperature
NEC: Necrotising enterocolitis
NST: Non-shivering thermogenesis
SSC: Skin-to-skin care
TI: Thermal imaging
WHO: World Health Organisation
